# Hypoxia induces resistance to ALK inhibitors in the H3122 non-small cell lung cancer cell line with an *ALK* rearrangement via epithelial-mesenchymal transition

**DOI:** 10.3892/ijo.2014.2574

**Published:** 2014-08-01

**Authors:** AKIHIRO KOGITA, YOSUKE TOGASHI, HIDETOSHI HAYASHI, SHUNSUKE SOGABE, MASATO TERASHIMA, MARCO A. DE VELASCO, KAZUKO SAKAI, YOSHIHIKO FUJITA, SHUTA TOMIDA, YOSHIFUMI TAKEYAMA, KIYOTAKA OKUNO, KAZUHIKO NAKAGAWA, KAZUTO NISHIO

**Affiliations:** 1 Department of Genome Biology, Kindai University Faculty of Medicine, Osaka-Sayama, Osaka 589-8511, Japan; 2 Department of Surgery, Kindai University Faculty of Medicine, Osaka-Sayama, Osaka 589-8511, Japan; 3 Department of Medical Oncology, Kindai University Faculty of Medicine, Osaka-Sayama, Osaka 589-8511, Japan

**Keywords:** non-small cell lung cancer, *EML4-ALK* rearrangement, ALK inhibitor, hypoxia, epithelial-mesenchymal transition

## Abstract

Patients with non-small cell lung cancer (NSCLC) with echinoderm microtubule-associated protein-like 4 (*EML4*)-anaplastic lymphoma kinase (*ALK*) rearrangements generally respond to ALK inhibitors such as crizotinib. However, some patients with *EML4-ALK* rearrangements respond poorly to crizotinib. Hypoxia is involved in the resistance to chemotherapeutic treatments in several cancers, and we investigated the association between the responses to ALK inhibitors and hypoxia. Sensitivity of the H3122 NSCLC cell line (*EML4-ALK* rearrangement) to ALK inhibitors (crizotinib or alectinib) was investigated during a normoxic or hypoxic state using an MTT assay. We found that the cell line was resistant to the inhibitors during hypoxia. Hypoxia mediated morphologic changes, including cell scattering and the elongation of the cell shape, that are characteristic of the epithelial-mesenchymal transition (EMT). A migration assay demonstrated that the number of migrating cells increased significantly during hypoxia, compared with during normoxia. Regarding EMT-related molecules, the expressions of slug, vimentin, and fibronectin were increased while that of E-cadherin was decreased by hypoxia. In addition, hypoxia inducible factor 1A-knockdown cancelled the hypoxia-induced EMT and resistance. Our findings indicate that hypoxia induces resistance to ALK inhibitors in NSCLC with an *EML4-ALK* rearrangement via the EMT.

## Introduction

Lung cancer is the leading cause of cancer-related death in the developed world (1). Non-small cell lung cancer (NSCLC) accounts for approximately 80% of lung cancers, and the prognosis of patients with advanced NSCLC remains very poor despite advances in treatment (2).

One promising treatment strategy involves the further subdivision of NSCLC into clinically relevant molecular subsets according to a classification schema based on specific so-called oncogenic driver mutations. These mutations occur in genes that encode signal proteins crucial for cellular proliferation and survival. Thus, cancer might rely on the expression of these single oncogenes for survival. This concept is also called oncogene addiction (3). The identification of epidermal growth factor receptor (*EGFR*) mutations as a type of oncogenic driver mutation in a subset of patients with NSCLC, coupled with the development of *EGFR* tyrosine kinase inhibitors (EGFR-TKIs), has opened new ways to treat this disease (4–7). Recently, a novel fusion transcript with transforming activity that is formed by the translocation of echinoderm microtubule-associated protein-like 4 (*EML4*) (2p21) and anaplastic lymphoma kinase (*ALK*) (2p23) has been described in a subset of NSCLCs (8). Although patients with *ALK* rearrangements respond dramatically to ALK inhibitors, as is seen in EGFR-TKIs for *EGFR*-mutated NSCLC, some of them are resistant to these inhibitors (9–11). Therefore, additional molecular mechanisms need to be identified to further improve the response.

Hypoxic cancer cells can be aggressive and exhibit metastatic phenotypes with lower sensitivity to apoptotic signals. In addition, hypoxia is involved in the resistance to chemotherapeutic treatments in several types of tumors, including *EGFR*-mutated NSCLC and EGFR-TKIs (12–14). However, the involvement of hypoxia in the resistance to ALK inhibitors in NSCLC with an *ALK* rearrangement remains unclear. In the present study, we investigated the influence of hypoxia on the sensitivity to ALK inhibitors in the H3122 NSCLC cell line with an *ALK* rearrangement.

## Materials and methods

### Cell culture and reagent

The H3122 cell line (NSCLC cell line with an *EML4-ALK* rearrangement) was maintained in RPMI-1640 medium with 10% FBS (Sigma-Aldrich, St. Louis, MO, USA). For the normoxic state, the cell line was maintained in a 5% CO_2_-humidified atmosphere at 37°C; for the hypoxic state, the cell line was maintained in 5% CO_2_-humidified 0.2% O_2_ at 37°C. Crizotinib and alectinib (ALK inhibitors) were purchased from Selleck Chemicals (Houston, TX, USA).

### Growth inhibition assay in vitro

The growth-inhibitory effects of crizotinib and alectinib were examined using a 3, 4, 5-dimethyl- 2H-tetrazolium bromide assay (MTT; Sigma-Aldrich), as described previously (15). The experiment was performed in triplicate.

### Migration assay

The migration assays were performed using Boyden chamber methods and polycarbonate membranes with an 8-μm pore size (Chemotaxicell; Kurabo, Osaka, Japan), as previously described (16). The membranes were coated with fibronectin on the outer side and were dried for 2 h at room temperature. The cells (2×10^4^ cells/well) were then seeded onto the upper chambers with 200 ml of migrating medium (RPMI containing 0.5% FBS), and the upper chambers were placed into the lower chambers of 24-well culture dishes containing 600 ml of RPMI with 10% FBS. After incubation for 48 h under a normoxic or hypoxic state, the media in the upper chambers were aspirated and the non-migrated cells on the inner sides of the membranes were removed using a cotton swab. The cells that had migrated to the outer side of the membranes were fixed with 4% paraformaldehyde for 10 min, stained with 0.1% crystal violet stain solution for 15 min, and then counted using a light microscope. The number of migrated cells was averaged from 5 fields per 1 chamber, and 3 chambers were used for each experiment. The experiment was performed in triplicate.

### Real-time reverse transcription PCR (RT-PCR)

One microgram of total RNA from cultured cell lines was converted to cDNA using the GeneAmp RNA-PCR kit (Applied Biosystems, Foster City, CA, USA). Real-time PCR was performed using SYBR Premix Ex Taq and Thermal Cycler Dice (Takara, Shiga, Japan), as described previously (16). The glyceraldehyde 3-phosphate dehydrogenase (GAPD, NM_002046) gene was used to normalize the expression levels in subsequent quantitative analyses. The experiment was performed in triplicate. To amplify the target genes encoding E-cadherin, vimentin, fibronectin, and slug (*E-cadherin, VIM, FN1* and *SLUG* gene), the following primers were used: *E-cadherin*-F, TTAAACT CCTGGCCTCAAGCAATC; *E-cadherin*-R, TCCTATCTT GGGCAAAGCAACTG; *VIM*-F, TGAGTACCGGAGACA GGTGCAG; *VIM*-R, TAGCAGCTTCAACGGCAAAGTTC; *FN1*-F, GGAGCAAATGGCACCGAGATA; *FN1*-R, GAGCT GCACATGTCTTGGGAAC; *SLUG*-F, ATGCATATTCG GACCCACACATTA; *SLUG*-R, AGATTTGACCTGTCTGC AAATGCTC; *GAPD*-F, GCACCGTCAAGGCTGAGAAC; *GAPD*-R, ATGGTGGTGAAGACGCCAGT.

### Antibody

Antibodies specific for AKT, phospho-AKT, ERK1/2, phospho-ERK1/2, PARP, cleaved PARP, caspase-3, cleaved caspase-3, E-cadherin, vimentin, slug, and β-actin were obtained from Cell Signaling (Beverly, MA, USA). An antibody specific for fibronectin was obtained from Abcam (Cambridge, UK). An antibody specific for hypoxia-inducible factor 1α (HIF1α) was obtained from Novus (Littleton, CO, USA).

### Western blot analysis

A western blot analysis was performed as described previously (15). Briefly, subconfluent cells were washed with cold phosphate-buffered saline (PBS) and harvested with Lysis A buffer containing 1% Triton X-100, 20 mM Tris-HCl (pH 7.0), 5 mM EDTA, 50 mM sodium chloride, 10 mM sodium pyrophosphate, 50 mM sodium fluoride, 1 mM sodium orthovanadate, and the protease inhibitor mix Complete™ (Roche Diagnostics, Basel, Switzerland). Wholecell lyses were separated using SDS-PAGE and were blotted onto a polyvinylidene fluoride membrane. After blocking with 3% bovine serum albumin in a TBS buffer (pH 8.0) with 0.1% Tween-20, the membrane was probed with the primary antibody. After rinsing twice with TBS buffer, the membrane was incubated with a horseradish peroxidase-conjugated secondary antibody and washed, followed by visualization using an ECL detection system and LAS-4000 (GE Healthcare, Buckinghamshire, UK). When the phosphorylation was evaluated in cells treated with an inhibitor, the cells were incubated under a normoxic or hypoxic state for 48 h and the inhibitor was added three hours before the sample collection.

### Short interfering RNA (siRNA) transfection

Cells were transfected with siRNA for *HIF1A* or a non-specific target (scramble) as follows: GGAAUUAACUCAGUUUGAACUAACU (si-HIF1A) and AAACCUUCAGACGUUAGUUUAUAGA for a scramble of HIF1A (si-Scr). siRNA transfection was performed using RNAiMAX (Invitrogen, Carlsbad, CA, USA) according to the manufacturer’s instructions, as previously described (17), and was allowed to proceed for 48–96 h before the growth-inhibitory test or the collection of the whole-cell extract. Knockdown was confirmed using western blot analyses.

### Statistical analysis

Continuous variables were analyzed using the Student’s t-test, and the results were expressed as the average and standard deviations (SD). The statistical analyses were two-tailed and were performed using Microsoft Excel (Microsoft, Redmond, WA, USA). P-values of <0.05 were considered statistically significant.

## Results

### Sensitivity to ALK inhibitors under a normoxic or hypoxic state

**Figure 1:**
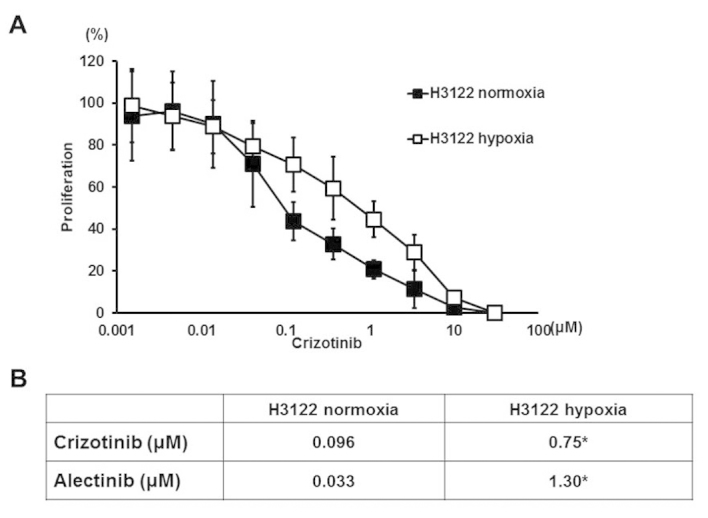
Sensitivity of the H3122 cell line to ALK inhibitors (crizotinib and alectinib). To examine the sensitivity of ALK inhibitors, we used an MTT assay. The experiment was performed in triplicate. (A) Growth inhibitory effect of crizotinib. The H3122 cell line was sensitive to crizotinib under a normoxic state, compared with a hypoxic state. The graphs, mean of independent triplicate experiments; error bars, SD. (B) IC_50_ of ALK inhibitors. The IC_50_ values of both inhibitors in the H3122 cell lines were significantly higher under hypoxia than under normoxia (crizotinib, ^*^P=0.028 and alectinib, ^*^P=0.0035). ^*^P<0.05.

To examine the sensitivity of the H3122 cell line to ALK inhibitors, we performed an MTT assay (Fig. 1A). Under a normoxic state, the 50% inhibitory concentration (IC_50_) of crizotinib and alectinib was 0.096 and 0.033 μM, respectively (Fig. 1B). Under a hypoxic state, however, the IC_50_ was 0.75 and 1.30 μM, respectively. These results indicate that hypoxia induces resistance to ALK inhibitors in the H3122 cell line.

### Effect of hypoxia on AKT and ERK signals and apoptosis in the H3122 cell line

**Figure 2:**
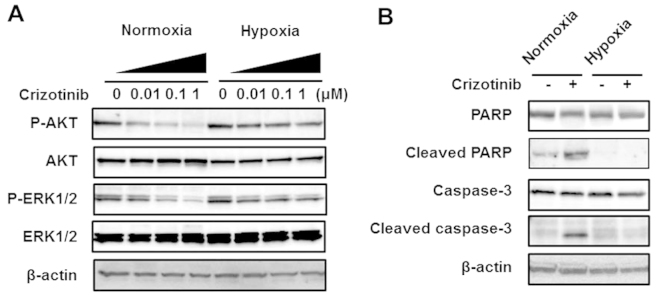
Western blot analyses of a downstream signal and apoptosis-related molecules in the H3122 cell line. (A) Downstream signal. The cells were incubated under normoxia or hypoxia for 48 h, then crizotinib was added three hours before the sample collection. The concentrations of crizotinib were 0, 0.01, 0.1 and 1 μM. The phosphorylation of AKT and ERK was dose-dependently reduced by crizotinib under normoxia. Under hypoxia, however, the phosphorylation was less reduced by crizotinib. β-actin was used as an internal control. (B) Apoptosis-related molecules. The samples were collected 48 h after DMSO (control) or crizotinib stimulation under normoxia or hypoxia. The concentration of crizotinib was 0.1 μM. The expression of both cleaved PARP and cleaved caspase-3 was elevated by crizotinib under normoxia, whereas neither expression level was elevated under hypoxia. β-actin was used as an internal control.

Next, to examine the effect of hypoxia on the downstream signal of ALK, we evaluated the phosphorylation of AKT and ERK under normoxic and hypoxic states. The cells were incubated under normoxia or hypoxia for 48 h, and the inhibitor was then added at the indicated concentrations three hours before the sample collection. The phosphorylation of AKT and ERK was dose-dependently reduced by crizotinib under a normoxic state. Under hypoxia, however, crizotinib reduced the phosphorylation to a lesser extent (Fig. 2A).

Furthermore, to evaluate apoptosis, western blot analyses for apoptosis-related molecules were performed. The samples were analyzed 48 h after DMSO (control) or crizotinib (0.1 μM) stimulation under normoxia or hypoxia. Under a normoxic state, the expressions of both cleaved PARP and cleaved caspase-3 were elevated by crizotinib, whereas the expressions were not elevated under hypoxia. These results suggest that the downstream signals are inactivated by crizotinib to a lesser degree and that crizotinib-induced apoptosis is inhibited under a hypoxic state.

### EMT of the H3122 cell line is mediated by hypoxia

**Figure 3:**
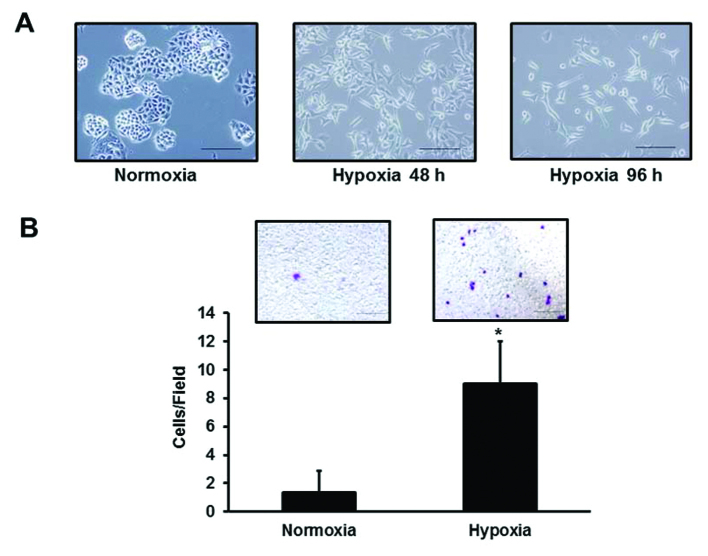
Morphologic change and migration of the H3122 cell line. (A) Morphologic change. Hypoxia time-dependently induced morphologic changes that are characteristic of the EMT, including cell scattering and an elongation of the cell shape. Scale bar, 20 μm. (B) Migration. The migration assays were performed using the Boyden chamber method. After incubation for 48 h under normoxia or hypoxia, the cells that had migrated to the outer side of the membranes were fixed and stained with crystal violet staining solution, then counted using a light microscope. The experiment was performed in triplicate. Under hypoxia, the number of migrating cells was significantly higher than that under normoxia (8.67±3.5 vs. 1.33±1.53/Field, ^*^P=0.026). Columns, mean of independent triplicate experiments; error bars, SD; ^*^P<0.05.

To investigate the mechanism of the resistance induced by hypoxia, the morphologic changes of cells subjected to hypoxia were observed. The morphology of the cells changed to a scattered pattern of spindle-shaped cells in a time-dependent manner (Fig. 3A). Then, to evaluate the migration ability, a migration assay was performed using the Boyden chamber method. Under hypoxia, the number of migrating cells increased significantly, compared with the situation under normoxia (8.67±3.5 vs. 1.33±1.53/Field, ^*^P=0.026) (Fig. 3B).

**Figure 4:**
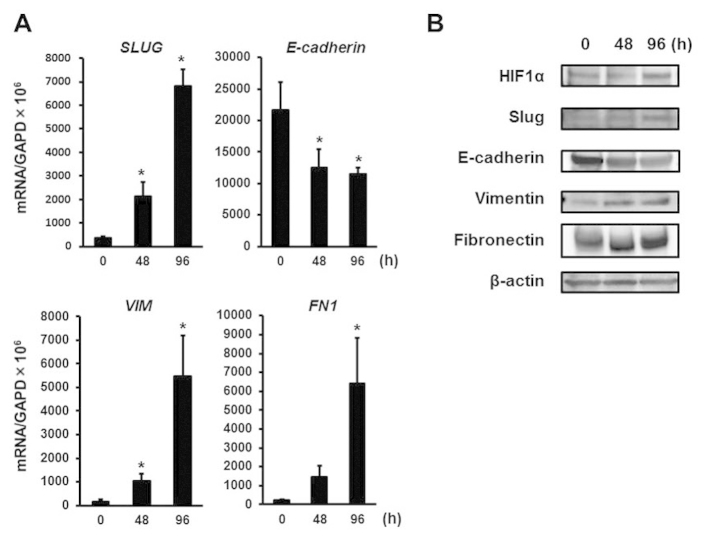
Real-time RT-PCR and western blot analyses for EMT-related molecules. (A) Real-time RT-PCR for EMT-related genes. To evaluate whether hypoxia mediates the EMT in H3122 cells, changes in the mRNA expression levels of EMT-related genes were evaluated using real-time RT-PCR. The experiment was performed in triplicate. Hypoxia upregulated *SLUG* mRNA expression (48 h, ^*^P=0.030 and 96 h, ^*^P=0.0053). *E-cadherin* mRNA expression was clearly downregulated under hypoxia (48 h, ^*^P=0.035 and 96 h, ^*^P=0.042). The expression of mesenchymal marker *VIM* and *FN1* mRNA was also upregulated (48 h, ^*^P=0.025 and ^*^P=0.079, respectively and 96 h, ^*^P=0.031 and ^*^P=0.044, respectively). Columns, mean of independent triplicate experiments; error bars, SD; ^*^P<0.05. (B) Western blot analyses for EMT-related molecules. Consistent with the mRNA changes, hypoxia time-dependently upregulated the protein expression of slug, fibronectin, and vimentin and downregulated the expression of E-cadherin in the cell line, along with HIF1α upregulation. β-actin was used as an internal control.

The EMT is characterized by an increase in cell scattering and an elongation of the cell shape (18,19). To evaluate whether hypoxia mediates the EMT in the H3122 cell line, changes in the mRNA expression levels of EMT-related genes were evaluated using real-time RT-PCR. Hypoxia time-dependently upregulated *SLUG* mRNA expression, which is considered to be a master regulator of the EMT (Fig. 4A). The downregulation of E-cadherin is also known to be a pivotal cellular event in the EMT (19,20). *E-cadherin* mRNA expression was clearly downregulated under hypoxia (Fig. 4A). The expression of mesenchymal marker *VIM* and the *FN1* mRNA was also upregulated (Fig. 4A). Consistent with the mRNA changes, hypoxia time-dependently upregulated the protein expression of slug, fibronectin, and vimentin and downregulated the expression of E-cadherin, along with HIF1α upregulation, which plays a central role in the hypoxic cellular responses (21) (Fig. 4B).

### HIF1A-knockdown effect on hypoxia-induced EMT and the resistance to ALK inhibitors

**Figure 5:**
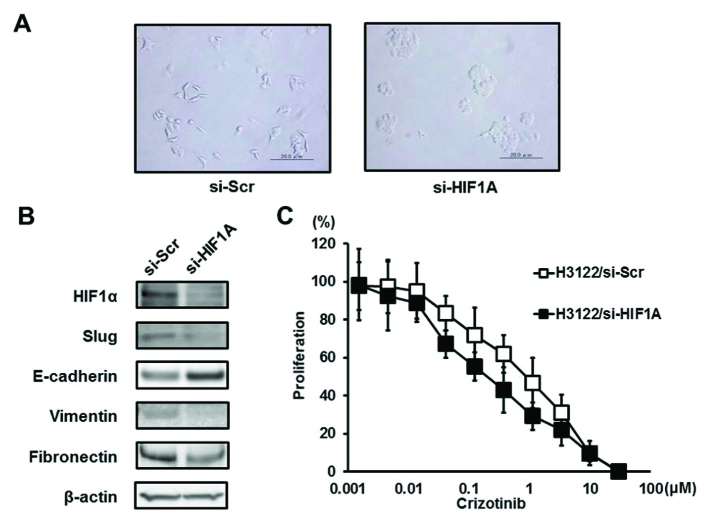
*HIF1A*-knockdown effect on the H3122 cell line under hypoxia. To investigate whether HIF1α was a key factor in hypoxia-induced EMT and resistance to ALK inhibitors, *HIF1A* was knocked down using specific siRNA (H3122/si-HIF1A) or a control (H3122/si-Scr). (A) Morphologic change. Both H3122/si-HIF1A and H3122/si-Scr cell lines were incubated for 48 h under a hypoxic state. *HIF1A*-knockdown abolished the hypoxia-induced morphologic changes (si-HIF1A), whereas the control-siRNA did not (si-Scr). Scale bar, 20 μm. (B) Western blot analyses for EMT-related molecules. Before the sample collection, both the H3122/si-HIF1A and the H3122/si-Scr cell lines were incubated for 48 h under hypoxia. *HIF1A*-knockdown cancelled the hypoxia-induced downregulation of E-cadherin and the upregulation of slug, vimentin, and fibronectin. β-actin was used as an internal control. (C) Growth inhibitory effect of crizotinib. To examine the sensitivity, we used an MTT assay during a hypoxic state. The experiment was performed in triplicate. Under hypoxia, the H3122/si-HIF1A cell line was more sensitive to crizotinib, compared with the H3122/si-Scr cell line. The graphs, mean of independent triplicate experiments; error bars, SD.

As a major transcription factor, HIF1α plays a central role in hypoxic cellular responses, and this transcription factor is reportedly related to the EMT (21,22). *HIF1A* was knocked down using specific siRNA to investigate whether it was a key factor in hypoxia-induced EMT and resistance to ALK inhibitors. *HIF1A*-knockdown abolished the hypoxia-induced morphologic changes (si-HIF1A), whereas the control-siRNA did not (si-Scr) (Fig. 5A). Similarly, *HIF1A-*knockdown abolished the hypoxia-induced downregulation of E-cadherin and the upregulation of slug, vimentin, and fibronectin (Fig. 5B). That is, *HIF1A*-knockdown cancelled the hypoxia-induced EMT, resulting in a mesenchymal-epithelial transition (MET). In addition, the resistance to crizotinib was abolished by *HIF1A*-knockdown (Fig. 5C). These findings indicate that HIF1α transcriptionally regulates the expressions of EMT-related molecules during hypoxia and is implicated in hypoxia-induced resistance to ALK inhibitors in the H3122 cell line.

## Discussion

The *EML4-ALK* rearrangement has been identified in 5–10% of NSCLC cases, and ALK inhibitors show marked antitumor effects in such tumors (8–11). However, some of these tumors are resistant to inhibitors, and acquired resistance to ALK inhibitors has already been found to limit the therapeutic potential of these agents (23–25); thus, an investigation of the mechanisms underlying such resistance is warranted. In this study, we found that hypoxia mediated the resistance to ALK inhibitors in the H3122 NSCLC cell line with an ALK rearrangement and that the resistance arose from hypoxia-induced EMT. To the best of our knowledge, this is the first report to show that hypoxia mediates the resistance to ALK inhibitors via EMT.

Several reports have discussed resistance to ALK inhibitors, such as secondary mutations in the ALK tyrosine kinase domain, the activation of other receptor tyrosine kinases (RTK), the EMT, and so on (23–27). Second-generation ALK inhibitors (i.e., alectinib and ceritinib) can be effective against resistant tumors with secondary mutations, and heat shock protein 90 inhibitors or the inhibition of other RTK signals can also overcome the resistance (24,25,28,29). The EMT is a cellular process of morphological change from an epithelial polarized shape to a mesenchymal fibroblastoid shape, in addition to accompanying behavioral changes such as enhanced mobility (18,19). Recently, several studies have reported that this phenomenon is strongly associated with cancer cell stemness (30,31) and resistance to drugs such as EGFR-TKIs (32–34). Our present study showed that hypoxia-induced EMT was associated with resistance not only to a first-generation ALK inhibitor, crizotinib, but also to a second-generation inhibitor, alectinib. Similarly, another study has demonstrated that the EMT mediates resistance to both first- and second-generation ALK inhibitors (27). In addition, our study showed that *HIF1A*-knockdown, which promoted MET, abolished the resistance. These findings suggest that EMT is associated with both first- and second-generation ALK inhibitors in NSCLC with an ALK rearrangement and that MET can cancel the resistance.

Hypoxic cancer cells can be aggressive and exhibit metastatic phenotypes with lower sensitivity to apoptotic signals (12). HIF1α is a key transcription factor that is induced by hypoxia (21), and it activates the transcription of genes implicated in tumor angiogenesis, cell survival, and resistance to chemotherapeutic drugs (12–14). Similarly, in our present study, activated AKT and ERK signals were less inhibited by crizotinib, and crizotinib-induced apoptosis was also inhibited under hypoxia. In addition, it has been reported that HIF1α stability induced by hypoxia promotes EMT via an EMT-triggering signal (i.e., TGFB and Notch), inflammation (i.e., TNFα, IL-1, and IL-6), and EMT transcriptional factors (i.e., twist, snail and slug) (22). In the present study, slug, which is one of the master regulators of EMT, was upregulated along with the upregulation of HIF1α during hypoxia, resulting in EMT and resistance to ALK inhibitors. *HIF1A-*knockdown downregulated slug expression, resulting in MET, which abolished the resistance to ALK inhibitors. Previous studies have shown that hypoxia mediates the resistance to EGFR-TKIs in *EGFR*-mutated NSCLC via an EGFR signal or cancer cell stemness (13,14). However, our study shows for the first time, the association between hypoxia-induced EMT and resistance to ALK inhibitors in NSCLC with an ALK rearrangement. Indeed, the involvement of EMT in acquired resistance to crizotinib in a clinically treated patient has been reported, with a tumor specimen exhibiting a large quantity of necrotic tissue (35). Necrosis is strongly related to hypoxia; therefore, this finding suggests that hypoxia can induce EMT. To overcome this resistance, combination with HIF inhibitors or anti-angiogenic therapies might be a promising strategy, and further research is needed.

In conclusion, we have found that hypoxia mediates the resistance to ALK inhibitors in the H3122 NSCLC cell line with an *ALK* rearrangement. The resistance comes from hypoxia-induced EMT, which can be overcome by the inhibition of HIF or anti-angiogenic therapies. Our findings provide novel insight into the resistance to ALK inhibitors in NSCLC with an ALK rearrangement.
